# Effects of maternal chlorpyrifos diet on social investigation and brain neuroendocrine markers in the offspring – a mouse study

**DOI:** 10.1186/s12940-015-0019-6

**Published:** 2015-04-02

**Authors:** Aldina Venerosi, Sabrina Tait, Laura Stecca, Flavia Chiarotti, Alessia De Felice, Maria Francesca Cometa, Maria Teresa Volpe, Gemma Calamandrei, Laura Ricceri

**Affiliations:** Department Cell Biology and Neuroscience, Istituto Superiore di Sanità, Rome, Italy; Department Veterinary Public Health and Food Safety, Istituto Superiore di Sanità, Rome, Italy; Department Drug Research and Evaluation, Istituto Superiore di Sanità, Rome, Italy

**Keywords:** Organophosphates, Prenatal exposure, Social responsiveness, Oxytocin, Vasopressin, Estrogen receptor alpha, Estrogen receptor beta, Oxytocin receptor, Vasopressin receptor 1a, Amygdala

## Abstract

**Background:**

Chlorpyrifos (CPF) is one of the most widely used organophosphate pesticides worldwide. Epidemiological studies on pregnant women and their children suggest a link between in utero CPF exposure and delay in psychomotor and cognitive maturation. A large number of studies in animal models have shown adverse effects of CPF on developing brain and more recently on endocrine targets. Our aim was to determine if developmental exposure to CPF affects social responsiveness and associated molecular neuroendocrine markers at adulthood.

**Method:**

Pregnant CD1 outbred mice were fed from gestational day 15 to lactation day 14 with either a CPF-added (equivalent to 6 mg/kg/bw/day during pregnancy) or a standard diet. We then assessed in the offspring the long-term effects of CPF exposure on locomotion, social recognition performances and gene expression levels of selected neurondocrine markers in amygdala and hypothalamus.

**Results:**

No sign of CPF systemic toxicity was detected. CPF induced behavioral alterations in adult offspring of both sexes: CPF-exposed males displayed enhanced investigative response to unfamiliar social stimuli, whereas CPF-exposed females showed a delayed onset of social investigation and lack of reaction to social novelty. In parallel, molecular effects of CPF were sex dimorphic: in males CPF increased expression of estrogen receptor beta in hypothalamus and decreased oxytocin expression in amygdala; CPF increased vasopressin 1a receptor expression in amygdala in both sexes.

**Conclusions:**

These data indicate that developmental CPF affects mouse social behavior and interferes with development of sex-dimorphic neuroendocrine pathways with potential disruptive effects on neuroendocrine axes homeostasis. The route of exposure selected in our study corresponds to relevant human exposure scenarios, our data thus supports the view that neuroendocrine effects, especially in susceptible time windows, should deserve more attention in risk assessment of OP insecticides.

## Background

An increasing body of data collected in the past decade supports the developmental neurotoxicity of organophosphate insecticides (OPs), and of the most widely applied of them, chlorpyrifos (CPF), in particular. Most non-agricultural uses of CPF such as residential control of insect pests were phased out in the United States in 2001 [1] and in the European Union (EU) in 2005 [2]. Meanwhile, CPF remains by far the most used OP insecticide in agriculture in US and Europe today [3] and exposure of pregnant women, infants, and children in the general population to CPF is ubiquitous and occurs primarily through the diet [4].

Several epidemiological studies indicate that developmental OP exposure may affect children’s neuropsychological maturation [5]. In a New York newborn cohort, in utero exposure to CPF from household use induced delays in cognitive and psychomotor development [6] and these effects were not influenced by socio-economic factors [7]. Very low concentrations of CPF in cord blood were associated with 1.4% decrease of child’s IQ and 2.8% decrement of working memory [8], an effect more evident in males [9]. Significant associations between prenatal CPF exposure and structural changes in the developing human brain were also observed: high-exposed children showed frontal and parietal cortical thinning with disappearance or even reversal of the typical sex dimorphism in several cortical areas [10]. In addition, a positive association between autism spectrum disorders and prenatal residential proximity to overall organophosphates (3^rd^ trimester) and CPF (2^nd^ trimester) has been recently evidenced [11].

Both US and EU regulatory bodies have recently started a process for reconsideration of reference values for CPF in order to protect human health. Specifically the US EPA stated in its preliminary risk assessment that a coherent mode of action for CPF has not yet been elucidated to explain the effects reported for low dose exposure. In this framework several mechanisms have been proposed, suggesting that anticholinesterase toxicity might not be the only key event [12]. A specific neuroendocrine disruption that encompasses the brain–pituitary complex has been proposed so far for selected environmental contaminants such as bisphenol A, phthalates and polychlorinated byphenyls [13,14].

As for CPF, exposure to doses devoid of systemic toxicity during development has been shown to affect general biological processes such as cell cycle, apoptosis, DNA synthesis or oxidative stress processes [15,16]. In the central nervous system (CNS), several *in vivo* and *in vitro* studies evidenced that CPF affects neural cell replication and differentiation, resulting in immediate and delayed-onset changes in synaptogenesis, neurotransmitter release, expression of neurotransmitter receptors and of neuropeptides and their receptors, over and above the consequence of cholinesterase inhibition [17-19]. In rats, CPF affects neural systems beyond the cholinergic one, such as serotonergic and dopaminergic transmission, in a sex-dimorphic fashion [20]. Developmental CPF exposure causes long-term alterations in behaviors possibly related to these neural effects such as changes in cognitive performance in the 16-arm radial maze or increased risk-taking behavior, affecting both sexes to a different extent [21,22]. Response to novelty, anxiety and social behavior repertoire are major targets of CPF effects in mice [23-25]. In agreement with rat studies, CPF exposure affected in a different way male and female mice, and both magnitude and direction of the effects were dependent on dose and time windows of administration. Inefficacy of the selective serotonin re-uptake antagonist fluvoxamine, was observed in male and female prenatally exposed to CPF [25] substantiating the hypothesis that serotonergic transmission is targeted by CPF during CNS maturation [21,26]. Notably, perinatal exposure to CPF also affects the levels of the hypothalamic neuropeptides oxytocin (OXT) and vasopressin (AVP) in mice [27]. Hypothalamic neuro-peptides act as key regulators of anxiety, aggression as well as of various aspects of social behavior in mammals [28-30], moreover their release in specific brain regions such as hypothalamus and amygdala has a significant estrogenic and sex-dimorphic regulation [28,31-34].

In this framework, the consistent findings of alterations in social competencies and anxiety levels after developmental CPF exposure supports the hypothesis that CPF may interfere with maturation of sexually dimorphic neuroendocrine pathways in the developing CNS, in addition to its recognized cholinergic toxicity [13].

The goal of the present study was to evaluate the effects of developmental CPF exposure on social recognition and on gene expression of different neuroendocrine markers in hypothalamus and amygdala. To this aim, we attempted to mimic the most probable human perinatal exposure scenario by administering dietary CPF from gestational day 15 to lactation day 14 in CD-1 outbred female mice. In their offspring we evaluated the long term effects of such exposure on social recognition in both sexes at adulthood, analyzing, in parallel, the expression of estrogen receptor *(ER)α*, *ERβ*, *Oxt* and *Avp* genes (as neurophysin I and II precursors, respectively) as well as of their specific receptors oxytocin receptor (*OxtR)* and vasopressin receptor (*AvpR)1a* in two brain regions, hypothalamus and amygdala, which have a key role in neuroendocrine control of affective and social responses [29].

The effects of this dietary treatment schedule on acetylcholinesterase (AChE) activity in brain and blood was also assessed in dams at delivery and in offspring [postnatal day (PND) 0 and 14].

## Methods

### Animals and treatments

All experiments on animals were performed according to the European Community Council Directive 86/609/EEC and to Italian Legislation on Animal Experimentation (Legislative Decree 116/92). Experiments were effectively carried out according to the Animal Research: Reporting In Vivo Experiments (ARRIVE) guidelines for reporting animal studies.

Male and female mice of a Swiss-derived outbred strain (CD-1, Harlan, S. Pietro al Natisone, Italy), were housed in polycarbonate breeding cages with a 12–hr light-dark cycle (light on 20:00-8:00) and with free access to food and water. Females were inspected daily for the presence of the vaginal plug (Gestational Day, GD 0). The stud was removed 10 days after the discovery of the vaginal plug. On GD 15 females were randomly assigned to one of the two prenatal treatments [control standard (STD) diet (DP/1000, Altromin-Rieper, Vandoies-BZ), or CPF diet (CPF, SIGMA in the proportion of 57.15 mg CPF/kg of STD diet). This amount of CPF added to the diet corresponds to a dose of 6 mg/kg/bw/day according to a food consumption of approximately 5 g/day recorded in this strain of mice. CPF diet was administered to pregnant females from GD 15 until lactation/ PND 14.

The dose selected is higher than environmentally relevant exposure but lies within the range of doses used in rodent studies showing delayed neurobehavioral effects in the offspring in the absence of maternal or systemic toxicity. Specifically, the diet regimen was based on our previous studies indicating that the 6 mg/kg/day dose administered from GD 14 to 17 by oral gavage was safe with respect to reproductive performance of treated dams [pregnancy length, number of pups at delivery, sex ratio], and it does not induce overt toxic symptoms in dams or major effects on pup’s health parameters such as weight at delivery and impairment of growing rate [23]. All females consumed their assigned diets *ad libitum* and pellets were weighed every other day to estimate the amount of ingested food. As food consumption increases during the first week of lactation in comparison to pregnancy, lactating females assumed higher CPF levels than those received during pregnancy. However we maintained during lactation the same diet regimen administered during pregnancy as a previous study demonstrated that following a regimen similar to the present one (CPF at a dose of 5 mg/kg/day in the diet throughout gestation, delivery and lactation) non quantifiable CPF levels were found in the pups, while the estimate exposure derived from CPF concentration detected in the milk was around 0.025 mg/kg/day [35].

Sample size for behavioral tests was estimated to allow the detection of a standardized mean difference (delta effect size) = 1.2 between CPF and OIL groups, with a two-sided alpha = 0.05 and a power = 0.80. At these conditions, a minimum of n = 9 mice per group were needed. In the Open Field test, where male and female littermates were analyzed, at least 9 couples per treatment group were considered (for a total of ≥ 18 animals per group).

Twenty litters (10 STD and 10 CPF) were left undisturbed till weaning, and at this time males and females from each litter were caged separately. Animals from each litter were assigned to experimental procedures following this scheme: one male and one female were assigned to behavior analysis and one male and one female from each litter were assigned to neuroendocrine assessment at adulthood. Slight differences in sample size between experiments were due to technical problem during video-recording.

Fourteen (5 STD and 9 CPF) additional dams were assigned to the assessment of CPF effect on acetylcholinesterase (AChE) activity in brain and blood 24 h within delivery. AChE activity was further assessed on tissue samples on PND 0 (serum: 6 STD and 8 CPF; brain: 8 STD and 8 CPF) and on PND 14 (serum: 8 STD and 8 CPF; brain: 8 STD and 8 CPF) that were littermates of subjects undergoing behavioral and neuroendocrine assessments at adulthood.

### AChE enzymatic analysis

Mouse brains were removed from skull, weighed and homogenized (1 min) in cold 0.038 M Tris–HCl buffer, pH 7.6. Blood samples were collected centrifuged (1000 *g* at 4°C for 5 min) to separate plasma from red blood cells. Serum was collected and frozen for subsequent analysis.

AChE activity was measured by Ellman’s spectrophotometric method [36-38]: Acetylthiocholine 0.56 mM was used as substrate and incubation in 0.05 M sodium phosphate buffer, pH 7.2 lasted 30 min at 37°C. Aliquots of brain homogenates (100 μl diluted 1:5 for pups PND 0, 50 μl diluted 1:20 for pups PND 14 and 50 μl diluted 1:10 for females at delivery) were used for enzymatic analyses. Aliquots of serum (25 μl diluted 1:20 for pups PND 0 and diluted 1:80 for mothers and pups PND 14) were used for enzymatic analyses.

### Behavioral assessment

Males and females of both STD and CPF groups underwent behavioral testing on PND 70 during the dark phase of the cycle. Behavioral analysis was performed by observers blind to the treatment received by each animal.

### Open field

Animals underwent a single 20-min open-field test. 10 STD and 9 CPF from each sex were individually placed in the centre of a black plexiglas open-field arena (40 × 40 × 40 cm). A videocamera suspended over the arena was connected to a videotracking system (Ethovision, Noldus, NL) to record and analyze spontaneous behavior in the arena. The following variable were considered: distance moved in the arena to assess locomotor activity levels; time spent and number of visit in central and peripheral areas to assess preference for safer zone, and speed. Furthermore, we analyzed the time course of distance moved by dividing the 20 min total observation in four 5-min blocks.

### Social recognition

Males and females from each diet group underwent a social recognition test which was administered following two different protocols according to sex. As for males, the test consisted of repeated exposure to a con-specific kept under a small wire cage to prevent direct “aggressive” responses between the two males. As for females, we selected a direct free interaction protocol, since no aggressive responses occur during female-female mouse encounters. Direct free interaction protocols in female mice have greater resolution power since they allow to record not only behavioral responses but also ultrasonic vocalizations (that are elicited in the residential female by an unfamiliar female) [39-41]. Importantly, from our previous studies in developmental CPF exposures, we know that vocalization rates in such female task can be modified by prenatal CPF exposure [42]. This methodological choice should maximize the likelihood to detect effects of the CPF treatment in the two sexes; however it hinders direct comparisons between male and female performances because of different length of intertrial intervals (15 vs 45 min) affecting retention performances and different presentation of social stimuli (wire-caged male vs free interacting female).

### Males

19 male mice (STD n = 9; CPF n = 10) were tested individually. The social recognition test took place on 2 days in a sound attenuated chamber. The social recognition task was preceded by a habituation session: each experimental subject was introduced for 10 min in the experimental cage enriched with a small round wire cage; after this period experimental mice underwent the social recognition test: Test (S1) - a stimulus mouse, unfamiliar con-specific of the same sex and age, was introduced for 5 minutes inside the home cage enclosed in the small, round wire cage; Retest same (S2) - after an inter-trial interval of 15 min the S1 stimulus mouse is re-introduced in the home cage enclosed in the wire cage for 5 min; Retest different (S3) - after an inter-trial interval of 15 min, a novel stimulus mouse was enclosed in the same wire cage and introduced in the home cage for 5 min. The entire social recognition test was videorecorded and later analyzed by an experimenter blind to treatment condition of the male “The Observer”, a software system for collection and analysis of behavioral data (Noldus, NL). During video-recorded sessions (S1, S2, S3), the following behavioral responses were considered (frequency, duration and latency): social investigation, sniffing the round wire cage and the stimulus animal; rearing, standing on hind legs; grooming, self-cleaning (wiping, licking, combing or stretching of any part of the body).

### Females

18 female mice (STD n = 9; CPF n = 9) were individually housed in standard cages for 3 days and thus considered as resident females. Additional untreated females (n = 3) (of comparable age and body weight) socially housed served as test partners. The social recognition test took place on the following day in a sound attenuated chamber. Test (S1) - a female partner is introduced in the home cage of the isolated resident for 3 min and after that returned to its home cage; Retest Same (S2) - 45 min after the test, the same female partner is re-introduced in the home cage of the resident for 3 min; Retest Different (S3) - 45 min after the Retest Same, a novel partner is introduced for 3 min in the cage of the resident. For each resident, partners used in S2 and S3 phases were never drawn from the same social group to provide the maximum olfactory novelty during the S3 phase. During the three experimental phases number of ultrasonic vocalizations (USVs) was assessed using a ultrasonic microphone (Avisoft UltraSound-Gate condenser microphone capsule CM16, Avisoft Bioacoustics, Germany) sensitive to frequencies between 10-180 kHz and suspended 10 cm above the cage. Vocalizations were recorded using an Avisoft Recorder (Version 3.2). Settings included sampling rate at 250 kHz; format 16 bit. For acoustical analysis, recordings were transferred to Avisoft SASLab Pro (Version 4.40) and a fast Fourier transformation (FFT) was conducted. Spectrograms were generated with an FFT-length of 512 points and a time window overlap of 75% (100% Frame, Hamming window). The spectrogram was produced at a frequency resolution of 488 Hz and a time resolution of 1 ms. A lower cut-off frequency of 15 kHz was used to reduce background noise outside the relevant frequency band to 0 dB. Call detection was provided by an automatic threshold-based algorithm and a hold-time mechanism (hold time: 0.005 s). An experienced user checked the accuracy of call detection, and obtained a 100% concordance between automated and observational detection. The broad range for USV recording allowed detecting all USVs emitted but did not provide information about possible different meanings of such USVs (e.g. appetitive versus aversive meaning). During the test, the 3 min female–female encounters were also recorded by a videocamera placed 12 cm far from the cage. Videotapes were later analyzed by an experimenter blind to treatment condition of the resident females using “The Observer”, a software system for collection and analysis of behavioral data (Noldus, Wageningen 6700 AG, 177 The Netherlands). During video-recorded sessions (S1, S2, S3), frequency and duration of the following behavioral responses emitted by the resident female were collected: – exploring: moving around the cage, sniffing the physical environment, rearing; – social investigation: sniffing any part of the partner’s body; – grooming: self-cleaning (licking, combing and stretching any part of its own body).

### Neuroendocrine assessment

On PND 70, behaviorally-naïve littermates of mice undergoing behavioral assessment were sacrificed and brain dissected (STD n = 5; CPF n = 5 for each sex). Brains were removed from skulls and ventral surface was exposed and hypothalamus removed. Amygdala nuclei were removed from a brain slice (3 mm tick, fifth-eighth coronal channel) obtained by a rodent brain matrix for adult mice (ASI Instruments, RBM 2000C, MI, USA). Hypothalami and amygdalae were flash frozen in liquid nitrogen and stored at -80°C until use. Following homogenization by the Miccra D-1 homogenizer (ARTmoderne Labortechnik, Müllheim, Germany), total RNA was extracted using the RNAeasy Mini Kit (QIAGEN, Hilden, Germany). RNA quantity was assessed with NanoDrop (NanoDrop, Wilmington, DE, USA) whereas integrity was evaluated by a 1% agarose gel electrophoresis. One μg of total RNA from each sample was retrotranscribed to cDNA using the cDNA Synthesis Kit (Quantace, London, UK). Specific primers for *Oxt* (Neurophysin I OXT carrier protein), *Avp* (Neurophysin II AVP carrier protein), *OxtR*, *AvpR1A*, *ERα*, *ERβ*, as well as for glyceraldehyde-3-phosphate dehydrogenase (*Gapdh*) and *β*-actin (*Actb)*, as reference genes, were designed using the Primer-BLAST web application (www.ncbi.nlm.nih.gov/tools/primer-blast) and purchased from Invitrogen (Life Technologies, Paisley, UK). Primers sequences are listed in Table 1. The SensiMix *Plus* SYBR (Quantace) was used to perform Real-time PCR assays running reactions on a Stratagene MX5005P thermocycler instrument. Experiments were performed in duplicate on 96 well PCR plates. The thermal program was as follows: 1 cycle at 94 for 10 minutes; 40 cycles at 94°C for 10 seconds, 58°C for 10 seconds and 72°C for 10 seconds; 1 dissociation cycle from 55 to 94°C to verify amplification products. Evaluation of *Gapdh* and *Actb* stability was performed with BestKeeper [43]. In hypothalamus the two reference genes were highly correlated among each other (Pearson coefficient r = 0.930; p-value = 0.001) and the calculated BestKeeper index presented a standard deviation (SD) = 0.90. By contrast, in amygdala, the BestKeeper index had a SD = 1.2 with a lower correlation between *Gapdh* and *Actb* (r = 0.727; p-value = 0.001). Since *Actb* had the lower SD (0.65 vs 0.91 for *Gapdh*), only this one was used as reference gene in both tissues to calculate mean relative expression values + SEM.

**Table 1:** **Sequences of the specific primers used in Real-time PCR**

***Gene***	***RefSeq accession***		***Sequence 5’ to 3’***	***Amplicon lenght***
*GAPDH*	NM_008084.2	fw	ACCCAGAAGACTGTGGATGG	172 bp
		rev	ACACATTGGGGGTAGGAACA	
*ACTB*	NM_007393.3	fw	TTGCTGACAGGATGCAGAAG	238 bp
		rev	GAAAGGGTGTAAAACGCAGC	
*OXT (Neurophysin I)*	NM_011025.4	fw	CCCCAGTCTCGCTTGCTGCC	191 bp
		rev	CGAAGCAGCCCAGCTCGTCC	
*AVP (Neurophysin II)*	NM_009732.1	fw	AGCCCGAGTGCCACGACGGT	146 bp
		rev	GGGCTTGGCAGAATCCACGGAC	
*OXTR*	NM_001081147.1	fw	ACCATCCTGCTCGCCTGGGT	197 bp
		rev	ATGCCCTCTGGGGCTCTCCG	
*AVPR1a*	NM_016847.2	fw	CCCTTGCCTCGGACAAGCCG	226 bp
		rev	GTGGGAGCCTCGCGGGAAAC	
*ESR1 (ERα)*	NM_007956.4	fw	CTCAACCGCCCGCAGCTCAA	223 bp
		rev	CCCCCAGGCTGTTGGCACTG	
*ESR2 (ERβ)*	NM_207707.1	fw	ACCCTCACTGGCACGTTGCG	204 bp
		rev	GGCTTGCGGTAGCCAAGGGG	

### Statistical analysis

Normality distribution of data was evaluated by means of Shapiro-Wilk test; homogeneity of variance was assessed by means of Levene Test; Greenhouse-Geisser correction was applied to take into account potential violation of the assumption of Compound Symmetry/Sphericity in the case of repeated measures analyses. AChE data were analyzed by unpaired Student’s t-Test. On open field data, analysis of variance (ANOVA) included prenatal treatment (2 levels) as between-litter fixed factor, sex as within-litter factor and 5 min-block as repeated measures (4 levels). As for social recognition test the model of analysis applied in each sex included prenatal treatment (2 levels) as between-subject fixed factor, and session as repeated measures (3 levels). Posthoc comparisons were performed using Tukey’s test, which can be applied also in the absence of significant ANOVA results [44].

Due to the low sample size, data on gene expression were analyzed by Kruskal-Wallis ANOVA, comparing the four subgroups (CPF males, CPF females, OIL males, and OIL females) with respect to each brain area separately. Chi-square partitioning of the overall Kruskal-Wallis chi-square was then performed to assess main effects of treatment (2 levels, 1 degree of freedom, df) and sex (2 levels, 1 df), and their interaction (1df). Posthoc Mann-Whitney tests were performed in case of significant interactions.

## Results

### Reproductive performance and AChE activity

Confirming our previous data, analysis of body weight data, litter size and sex ratio did not show any significant effects of CPF supplemented diet on the offspring (data not shown).

When measured on the day of delivery, dietary CPF exposure induced 80% inhibition of serum AChE in the dams (T test p < 0.01) but not of brain AChE. As for the offspring, only a significant but very slight inhibition (about 10%) was found at PND 14 for blood AChE, while no CPF effects was found for brain AChE either at birth or at PND 14 (see Table 2).

**Table 2:** **AChE activity following developmental CPF exposure**

			**Standard Diet**	**CPF**	
Dams	Day of delivery	Serum	5689 ± 1268	1159 ± 240	(**)
		Brain	14547 ± 437	14311 ± 1172	
Pups	PND 0	Serum	1713 464	1629 377	
		Brain	1671 ± 162	1789 ± 175	
	PND 14	Serum	7170 ± 561	6445 ± 300	
		Brain	14869 ± 1074	15537 ± 1188	

Data are mean value (nmol/min/gr of tissue) ± standard deviation; PND postnatal day; (**) p <0.01 following T-test.

### Open field

**Figure 1:**
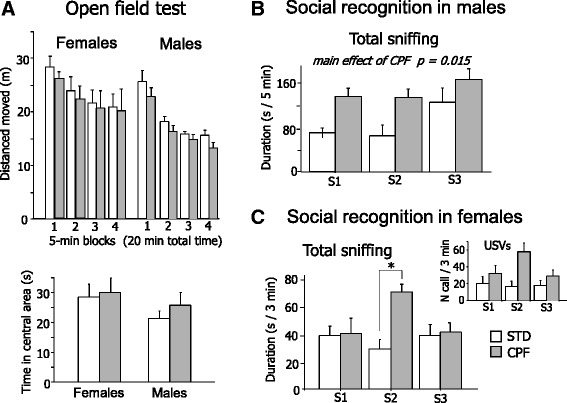
**Long-term behavioral effects of developmental exposure to CPF. A)** Open field test on PND 70. Upper panel: Total distance moved throughout the 20 min: both sexes and both treatments showed the expected progressive decrease of locomotor activity. Lower panel: Time spent in the central area of the arena (an index of anxiety); data are mean values + SEM, (STD: n = 10; CPF: n = 9 in each sex group. **B)** Male social recognition test. CPF males showed a significant higher level of social investigation (total sniffing) towards mouse partners than STD mice. S1 session 1 (5-min exposure to a partner; S2 session 2 (5-min exposure to the same partner ); S3 session 3 (5-min exposure to a novel partner). Sessions are spaced by a 15-min intertrial interval (ITI). Data are mean values + SEM, (STD: n = 9; CPF: n = 9); **C)** Female social recognition test. Larger graph: CPF females show increased social investigation (total sniffing) only during S2. Smaller graph: number of ultrasonic vocalizations (USVs) emitted by the resident female during the test, showing a profile across sessions comparable to sniffing response. S1 session 1 (3-min exposure to a partner); S2 session 2 (3-min exposure to the same partner); S3 session 3 (3-min exposure to a novel partner). Sessions are spaced by a 45-min intertrial interval (ITI). Data are mean values + SEM, (social investigation, STD: n = 9; CPF: n = 9; USV, STD: n = 4; CPF: n = 6). * p < 0.05.

Developmental exposure to CPF did not affect spontaneous behavior in the open field arena. No significant differences between STD and CPF mice were found either on total distance moved or on time spent in the central area (an index of anxiety). Habituation profile was evident in all animals [F (3, 51) = 38.27 p < 0.01] and not affected by CPF exposure. Only a main effect of sex was found [F (1, 17) = 0.021 p < 0.01], indicating a higher level of locomotion in females (see Figure 1).

### Social recognition

#### Males

**Figure 2:**
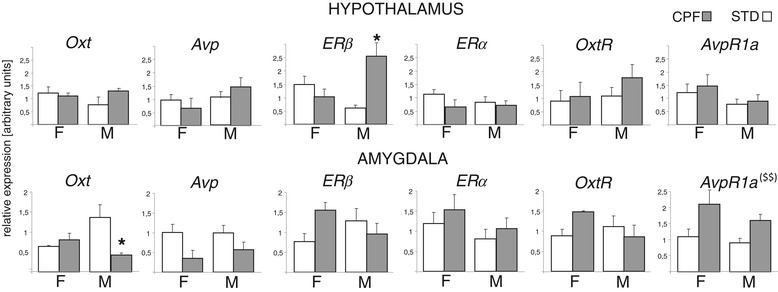
**Gene expression (mRNA levels) of neuroendocrine markers in hypotalamus (upper panel) and amygdala (lower panel) at PND 70.** *Oxt* neurophysin I, oxitocin; *Avp* neurophysin II, vasopressin; *ERβ* Estrogen Receptor beta; *ERα* Estrogen Receptor alpha; *OxtR* oxytocin receptor; *AvpR1a*, Vasopressin receptor 1a; * p < 0.05; ^($$)^ main effect of CPF treatment p < 0.01. Data are expressed as relative expression values (*Actb* as reference gene) + SEM.

CPF male mice showed higher social investigation (total sniffing) in each session of the test than STD mice [F (1, 17) = 8.42 p = 0.001]. As expected in the third session of the social recognition test both CPF and STD showed a renewal of interest towards the unfamiliar mouse [F (2, 34) = 12.78 p < 0.001] (see Figure 2A).

#### Females

As for social investigation duration, a main effect of CPF [F (1, 15) = 5.93 p = 0.028] and a treatment x session interactions [F (2, 30) = 3.79 p = 0.034] were observed. CPF females showed increased social investigation only during the second exposure to the same partner (S2) (p < 0.01, after post hoc comparisons) (Figure 2B) and did not show enhancement of social investigation of the novel partner during S3. USV emission, normally emitted during a female-female social encounter in a resident-intruder paradigm [41], follows exactly the same profile of social investigation throughout the three sessions of the test.

### Expression of neuroendocrine markers in Hypotalamus and Amygdala Hypothalamus

No differences between CPF and controls were evident for mRNA expression of *Oxt*, *OxtR*, *Avp*, *AvpR1a*, and *ERα*.

By contrast, *ERβ* was significantly increased only in CPF exposed males (see Figure 2) [sex x treatment interaction Chi-Square (1) = 6.2960, p = 0.0121; Mann-Whitney test CPF males vs STD males p = 0.02 ]. For *Avp* (neurophysin II) expression, only a main effect of sex [Chi-Square (1) =8.652 p = 0.03] was detected.

#### Amygdala

In the amygdala, CPF significantly decreased level of *Oxt* expression in males [sex x treatment interaction Chi-Square (1) = 7.,0003, p = 0.0081; Mann-Whitney test CPF males vs STD males p = 0.02]. A CPF-induced increase was found for *AvpR1a* expression in both sexes [main effect of treatment Chi-Square (1) = 7.3338, p = 0.0067] (see Figure 2).

## Discussion

In the absence of brain AChE inhibition, dietary CPF exposure throughout pregnancy and lactation modifies social behavior patterns and mRNA expression of underlying neuroendocrine markers in the adult offspring. Notably, CPF effects are different in the two sexes, with males more affected than females.

Behavioral responses in the two sexes cannot be directly compared in the present study, due to the different task (as well as different inter trial interval durations) applied. However it is noteworthy that CPF males are affected throughout all the sessions of the social recognition test, an effect more evident during the first two session of the test. CPF enhanced social response also in females, but with a different pattern: enhanced response was evident only during the second exposure to the same partner (just when habituation, i.e. a decrease, would be expected), a peculiar profile confirmed by USV emission rates. These behavioral data confirm the results of the prenatal or neonatal exposure protocols where CPF treatment induced enhanced response towards social salient stimuli (e.g. enhancement of aggressive behavior observed in adolescent males and of social and maternal responsiveness in females, [23,42]). Thus altered responsiveness to social cues appears as a hallmark of developmental exposure to CPF in mice, regardless exposure window selected.

As for neuroendocrine markers, both hypothalamus and amygdala appear as sensitive target areas for CPF exposure: as for males, CPF increased levels of *ERβ* expression in hypothalamus and decreased *Oxt* expression in amygdala; CPF also increased *AvpR1a* expression in amygdala in both sexes.

The sex- and region-specific effects of developmental CPF on *ERβ* are suggestive of mechanistic hypothesis to be further explored. The observation that CPF significantly induced *ERβ* expression in the hypothalamus and not in the amygdala suggests that CPF does not act as a general estrogen-like compound globally up-regulating *ERβ*. The selective CPF effect on *ERβ* in the absence of effect on *ERα*, could be explained by the existence of different ontogenetic expression patterns of *ERα*/*ERβ* transcripts [45,46]. Similar mechanisms could be responsible for the sexual dimorphic CPF effects, also considering the morphogenetic role of estrogen and ERβ in sexual differentiation and development of the brain [47].

CPF also impacts on *Oxt* and *AvpR1a* expression in amygdala. This interference has to be interpreted together with behavioral results. Of note the enhanced investigative responses to unfamiliar social stimuli observed in CPF-exposed mice (more evident in males) is associated with a decrease of *Oxt* expression in amygdala in males. Intriguingly, the behavioral profile of mice carrying deletion of the *Oxt* gene [28] also display impairments in habituation to a familiar partner whereas lack of *ERβ* gene had no consequences on social recognition in either male or female mice [48].

All together, these data confirm our previous hypothesis that exposure to CPF leads to inappropriate processing of the salience of diverse social cues [49]. Such hypothesis also fits well with the growing body of evidence from animal and human studies that implicates amygdalar OXT in the emotional processing of social stimuli and activation of the appropriate response (i.e. affiliation or aggression) [50,51], indeed, our data support the idea that amygdalar *Oxt* and *AvpR1a* upregulation represents the focal CPF-induced change underlying behavioral alterations.

This is the first *in vivo* study exploring potential adverse effects of CPF on molecular endpoints in amygdala. Future studies should focus on the links between the pattern of neuroendocrine effects observed in amygdala with selected downstream pathways known to be modulated by hypothalamic neuropeptides [52] and involved in the modulation of either the reward or the affective value of social cues. Those links likely involve neurotransmitter systems targeted by prenatal CPF, including serotonin [20,25,53,54] noradrenaline and dopamine [55,56].

A limited number of evidence is so far available exploring the endocrine disrupting potential of CPF, primarily exploring *in vitro* methods. CPF alters gonadotropin-releasing hormone biosynthesis in GT1-7 hypothalamic cell lines [57]; both anti-androgenic [58] and estrogenic effects, inducing growth in breast cancer cells [59,60], have been observed. Interestingly, still *in vitro* data from a wide screening study using cancer fibroblast cells (MCF-7BUS) evidenced that CPF selectively increases *ERβ*, but not *ERα* gene expression [61]. Our study confirms for the first time in vivo that *ERβ* is a target of CPF endocrine disrupting activity.

Interestingly, data from AChE in dams (actually receiving the CPF supplemented diet only for five days, during late pregnancy) show that opposite to a 80% reduction of serum AChE activity, no reduction of brain AChE activity was detected. These data partially confirm the order of magnitude of inhibition “ plasma > RBC > brain” developed by the PBPK/PD model developed for CPF in rat and humans, with inhibition of brain AChE occurring only at doses of 10 mg/Kg or higher [62]. Other rat data indicate marked brain AChE inhibition following CPF dietary exposure in adult rats, but only after chronic dietary exposures (e.g. 21 weeks [63]; 6-12 months [64]).

## Conclusions

The route of exposure selected in our study corresponds to relevant human exposure scenarios since it assures continuous levels of exposure from pregnancy to lactation. It is worth noting that, at variance from previous findings showing small but significant brain AChE inhibition in the offspring 4 and 24 hrs following administration of CPF [65], we did not find any evidence of brain AChE inhibition either at birth and at day 14 in the offspring. Thus, our findings support the view that mechanisms underlying developmental neurotoxicity of CPF are more complex and possibly independent from its anticholinergic activity.

Whereas it is clear that AChE inhibition is not the most sensitive endpoint, the spectrum of effects reported *in vitro,* in animal models and in humans coherently supports that endocrine effects, especially in susceptible time windows, should deserve more attention in the risk assessment of OP insecticides for a twofold reason. On one side, CPF neuroendocrine effects on *Oxt* and *AvpR1a* are extremely relevant since these pathways are implicated in several affective and emotional behaviors extending to psychopathology [29]. On the other, early alteration of physiological *ERβ* expression pattern could be associated with specific prenatal/perinatal morphogenetic pathways (e.g. in *ERβ -/-* mice abnormal migration of cortical neurons to upper *laminae* leads to permanent alteration in cortical organization [66,67]) which could have permanent consequences on brain development and functions, in line with child cohort studies reporting structural changes in developing brains [10].

## Abbreviations

AChE: Acetyl-cholinesterase
Actb: β actin
Avp: Vasopressin
AvpR1a: Vasopressin receptor 1a
CPF: Chlorpyrifos
CNS: Central Nervous System
ER: Estrogen receptor
FFT: Fast Fourier transformation
Gadph: Glyceraldehyde-3-phosphate dehydrogenase
GD: Gestational day
OPs: Organophosphate insecticides
Oxt: Oxytocin
OxtR: Oxytocin receptor
PND: Postnatal day
STD: Control standard diet
S1: Social recognition test
S2: Social recognition retest-same
S3: Social recognition retest-different
USV: Ultrasonic vocalization
